# Sarcosine, Trigonelline and Phenylalanine as Urinary Metabolites Related to Visceral Fat in Overweight and Obesity

**DOI:** 10.3390/metabo14090491

**Published:** 2024-09-10

**Authors:** Aline Maria Cavalcante Gurgel, Aline Lidiane Batista, Diogo Manuel Lopes de Paiva Cavalcanti, Alviclér Magalhães, Denise Engelbrecht Zantut-Wittmann

**Affiliations:** 1 Department of Biological and Health Sciences, Medical Course at the Federal University of the Semi-Arid, Mossoró 59625-900, RN, Brazil; aline.gurgel@ufersa.edu.br (A.M.C.G.); aline.bastista@ufersa.edu.br (A.L.B.); diogo.cavalcanti@ufersa.edu.br (D.M.L.d.P.C.); 2 Institute of Organic Chemistry, Federal University of Rio de Janeiro, Rio de Janeiro 21941-909, RJ, Brazil; alvicler@gmail.com; 3 Endocrinology Division, Department of Internal Medicine, School of Medical Sciences, University of Campinas, Campinas 13083-887, SP, Brazil

**Keywords:** obesity, metabolome, visceral fat

## Abstract

The objective of the present study is to analyze the urinary metabolome profile of patients with obesity and overweight and relate it to different obesity profiles. This is a prospective, cross-sectional study in which patients with a body mass index (BMI) ≥25 kg/m were selected. Anthropometric data were assessed by physical examination and body composition was obtained by bioimpedance (basal metabolic rate, body fat percentile, skeletal muscle mass, gross fat mass and visceral fat). Urine was collected for metabolomic analysis. Patients were classified according to abdominal circumference measurements between 81 and 93, 94 and 104, and >104 cm; visceral fat up to 16 kilos and less than; and fat percentiles of <36%, 36–46% and >46%. Spectral alignment of urinary metabolite signals and bioinformatic analysis were carried out to select the metabolites that stood out. NMR spectrometry was used to detect and quantify the main urinary metabolites and to compare the groups. Seventy-five patients were included, with a mean age of 38.3 years, and 72% females. The urinary metabolomic profile showed no differences in BMI, abdominal circumference and percentage of body fat. Higher concentrations of trigonelline (*p* = 0.0488), sarcosine (*p* = 0.0350) and phenylalanine (*p* = 0.0488) were associated with patients with visceral fat over 16 kg. The cutoff points obtained by the ROC curves were able to accurately differentiate between patients according to the amount of visceral fat: sarcosine 0.043 mg/mL; trigonelline 0.068 mg/mL and phenylalanine 0.204 mg/mL. In conclusion, higher visceral fat was associated with urinary levels of metabolites such as sarcosine, related to insulin resistance; trigonelline, related to muscle mass and strength; and phenylalanine, related to glucose metabolism and abdominal fat. Trigonelline, sarcosine and phenylalanine play significant roles in regulating energy balance and metabolic pathways essential for controlling obesity. Our findings could represent an interesting option for the non-invasive estimation of visceral fat through biomarkers related to alterations in metabolic pathways involved in the pathophysiology of obesity.

## 1. Introduction

The incidence of obesity has steadily increased, reaching 25.3% of the European population and 42% of the US population [1,2]. Similarly, in our country, the prevalence of obesity in adults increased from 11.8% in 2006 to 20.3% in 2019, with projected estimates of 68.1% for overweight, 29.6% for class I obesity and 9.3% for classes II and III obesity by 2030 [3].

There are several causes of obesity resulting from energy imbalance, in which calorie intake exceeds calorie expenditure, and these include biopsychosocial factors, genetics, physical activity, the consumption of chemical substances that disrupt the endocrine system, ethnicity and inadequate sleep, among others. Studies suggest that there is a significant relationship between obesity and the consumption of chemical additives in ultra-processed foods, as well as with environmental toxins such as bisphenol A (BPA) [4,5,6,7,8].

Several authors have investigated the relationship between different types of physical activity and obesity. According to Zhand et al., sedentary behavior is associated with an increased frequency of abdominal obesity, especially in women. In contrast, men who perform intense physical activities at work tend to have lower rates of obesity [9,10,11,12].

It is important to note that obesity is very complex and requires an approach that includes more than just diet and exercise. There is evidence to suggest that certain viruses contribute to the development of obesity, due to their influence on inflammation and metabolic pathways in the liver and adipose tissue [13,14].

Obesity is considered a heterogeneous disease and can be distinguished in various ways, according to body mass index (BMI), waist circumference, amount of visceral fat or total fat [15,16].

Perez-Campos et al. [17] suggest an anthropometric, clinical and metabolic classification of patients with obesity into the categories of metabolically healthy, metabolically abnormal obesity, metabolically abnormal normal weight and sarcopenic obesity. However, according to Lin et al. [18], obesity is better classified only by typically metabolic parameters, classified into four subtypes: metabolically healthy obesity (MHO), hypermetabolic obesity–hyperuricemia (HMO-U), hypermetabolic obesity–hyperinsulinemia (HMO-I) and hypometabolic obesity (LMO). MHO shows the lowest incidence of comorbidities, while HMO-U shows extremely high levels of uric acid. HMO-I is associated with insulin hypersecretion and an increased incidence of polycystic ovary syndrome. LMO shows a more affected glycolipid metabolism and a higher incidence of diabetes and metabolic syndrome.

Metabolomics has been used as a tool to better understand obesity, from diagnosis to treatment. It consists of the qualitative and quantitative detection of specific metabolites of various sizes present in body fluids or tissues. Recent studies have shown that detecting and quantifying these metabolites helps to understand the body’s physiology and the pathophysiology of many diseases, such as obesity [19,20].

Metabolomics can help identify different types of obesity and predict future risks, such as type 2 diabetes and mortality. Otosson et al. found that individuals with an “obese metabolome” despite having a normal BMI face elevated risk, revealing high-risk individuals who are not identified by weight alone. Metabolites such as glutamate, β-carotene, isoleucine, and kynurenine vary between obese and healthy metabolomes, which may indicate risk for diabetes. In addition, research shows that even with a not-very-high BMI, analyses of metabolites in blood and urine can reveal new subtypes of obesity associated with higher risks of heart disease, diabetes or metabolic syndrome [21,22,23,24,25,26,27,28,29].

Nuclear magnetic resonance (NMR) spectrometry is a non-invasive analytical method that identifies substances in complex mixtures and is used in metabolomic studies because it offers high reproducibility and the ability to identify metabolites regardless of the type of sample used [30]. This technique has been used to study several diseases. Qi et al. found that certain serum metabolites, particularly IDL and VLDL cholesterol, could discriminate obese individuals who would develop metabolic disorders from individuals who would remain healthy using NMR. Other studies have combined NMR-based metabolomics with machine learning to study metabolic targets involved in the progression of prediabetes to diabetes [31,32,33].

Some researchers have used magnetic resonance spectroscopy to evaluate the metabolite profile in the abdomen, hepatocytes and muscles of patients with different anthropometric measurements. They observed that visceral fat deposits had a significant influence on markers of dyslipidemia, such as serum triglyceride and HDL cholesterol levels. Others studied the deterioration of the metabolic profile and the deposition of ectopic fat induced by a high-fat diet in rats and found acetoacetate, N-acetylglycoprotein, lactate, VLDL/LDL and valine to be possible biomarkers [34,35,36].

Serum and urinary metabolomics have been used to assess the association between obesity and metabolic dysregulation. In patients with obesity with or without metabolic syndrome, it has been possible to detect a series of altered metabolites compared to normal-weight patients. Among the metabolites altered in plasma are lactate, acetoacetate, N-acetylglycoprotein, lipids, alanine and branched-chain amino acids (BCAA) [37,38].

Studies of urine samples have detected an association between muscle metabolism markers such as ketoleucine, ethanolamine and 3-methylhistidine and higher body weight in patients with obesity [39]. Other studies have shown that p-cresol sulfate, trimethylamine N-oxide, and urinary aromatic amino acids could be associated with metabolic dysregulation [40].

Thus, metabolomics can help to understand the various phenotypes of obesity [41]. Thus, the main objective of this study is to analyze the urinary metabolome profile of patients with obesity and overweight and relate it to anthropometric and biochemical characteristics. Secondly, a metabolic evaluation of the different types of obesity and overweight will be carried out.

## 2. Materials and Methods

### 2.1. Study Design and Patients

This is a cross-sectional study in which 75 patients with a body mass index (BMI) ≥ 25 kg/m^2^ were included, of whom 54 (72%) were female and 21 (28%) were male, with a mean age of 38.3 years, and with 19 (25.3%) overweight and 56 (74.7%) obese. Anthropometric data were assessed by physical examination and body composition obtained by bioimpedance. Urine was collected for metabolomic analysis and blood for metabolic analysis.

The patients were divided according to abdominal circumference measurements between 81 and 93, 94 and 104, or >104 cm; by visceral fat up to 16 kilos or over 16 kilos; by fat percentiles of <36%, 36–46% or >46%; and by BMI of 25–30, 30–35 or >35 kg/m^2^; and the metabolites selected from the metabolomic analysis were compared between the groups.

The inclusion criteria were overweight or obesity between the ages of 18 and 65. The exclusion criteria were pregnancy, uncontrolled hypertension, use of psychotropic drugs or antidepressants, previous cardiovascular events (angina, acute myocardial infarction, stroke) and age over 65 or under 18. Patients with decompensated hypertension and previous cardiovascular events were excluded because they were part of a phase of the study in which there was an intervention with contraindicated medications in these situations. The patients were included in the study after reading and signing an informed consent form. This study was approved by the local ethics committee under number 40678120.0.0000.5404.

### 2.2. Anthropometric Measurements

In all patients, the abdominal circumference (cm) was assessed with a measuring tape with a precision of 0.1 mm, with the patient in an orthostatic position, arms extended along the body and abdomen relaxed, using the midpoint between the lower costal margin and the iliac crest. The hip measurement (cm) was taken with the patient in an orthostatic position, with thighs together and arms alongside the body, at the largest part of the buttocks, located laterally to the pelvis. Height (cm) was measured using a stadiometer (accuracy 1 mm) with the patient in an orthostatic position, with bare feet together, and the posterior heel, pelvic girdle, shoulder girdle and occipital region in contact with the instrument. Weight (kg) was measured using a 100-gr precision digital scale, with the patient in an orthostatic position, bare-footed and wearing as little clothing as possible, in front of the measuring scale. BMI was calculated by dividing weight (in kg) by the square of height (in meters). The waist-to-hip ratio was calculated by dividing the waist circumference measurement in centimeters by the hip circumference measurement in centimeters.

### 2.3. Bioimpedance

The patients underwent bioimpedance with an octopolar InBody 120 device. The test was carried out on a scale with metal plates conducting a weak electric current through the body. The tetrapolar system had 8 electrodes and used 10 impedance measurements with two different frequencies (20 KHz, 100 KHz) in each of the 5 body segments (right arm, left arm, trunk, right leg and left leg). A segmental multifrequency bioimpedance meter, DSM-BIA, manufactured in 2019 by Ottoboni, was used. The test was carried out with the patient fasting, with no physical activity one hour before the test, in an orthostatic position, with no metal objects on the body and wearing light clothing. Bioimpedance assessed basal metabolic rate (BMR) (kcal), body fat percentile (%), skeletal muscle mass (kg), gross fat mass (kg) and visceral fat (kg).

### 2.4. Laboratory Assessment

Fasting blood glucose (reference value VR 70–99 mg/dL), glycated hemoglobin (VR < 6.5%), total cholesterol (VR < 200 mg/dL), HDL cholesterol (VR > 40 mg/dL), LDL cholesterol (VR < 100 mg/dL), triglycerides (VR < 150 mg/dL), uric acid (VR men < 7.5 mg/dL and women < 6.5 mg/dl), urea (VR < 40 mg/dl), creatinine (VR < 1.3 mg/dl), oxalacetic transaminase (TGO) (VR men up to 40 U/L; women < 31 U/L), pyruvic transaminase (TGP) (VR men up to 40 U/L; women < 31 U/L), thyroid stimulating hormone (TSH) (VR < 4.5 μUI/mL).

### 2.5. Acquisition of Urinary Samples, Measurement of Metabolites and Metabolome Analysis by Magnetic Resonance and Selection of Metabolites of Interest

After proper hygiene practices, urine samples were collected mid-stream after abstaining from eating, drinking, smoking or using oral hygiene products for at least 1 h and then placed in sterile tubes. The samples were centrifuged at 4 °C in 14000× *g*/RCF for 5 min, with 630 µL of the supernatant added to 70 µL of a stock solution of 1.5 M potassium phosphate buffer in deuterated water, containing 2 mM NaN_3_ and 5.8 mM TMSP, keeping the pH at 7.4. Subsequently, the samples were centrifuged with the buffer at 4 °C in 14, × *g*/RCF for 10 min, with 600 µL of the supernatant transferred to 5 mm magnetic resonance tubes with automated measurements at 25 °C. The ^1^H NMR spectra were measured with 128 scans, pre-saturating the H_2_O signal in a spectral window of 16 ppm (65,536 points) in the NMR equipment (500 MHz for ^1^H), using a waiting time of 1.5 s between each scan. The FID (Free Induction Decay) values obtained were processed, generating the spectra using a Fourier transform of 65,536 points and an exponential filter of 0.5 Hz strength to increase the signal-to-noise ratio. The phases of each of the spectra obtained were manually corrected and then the baseline was adjusted using an automatic function. The processed spectra were saved, read and used in Python routines to carry out multivariate analysis. The unsupervised PCA (principal component analysis) values were obtained using the processed spectra and subdivided into equal parts of 0.05 ppm (bins), which were duly integrated and used to make up the spreadsheet for the first statistical analysis. In this analysis, the regions between 4.65 and 4.95 ppm were disregarded as they were still signals from residual H_2_O.

**Figure 1:**
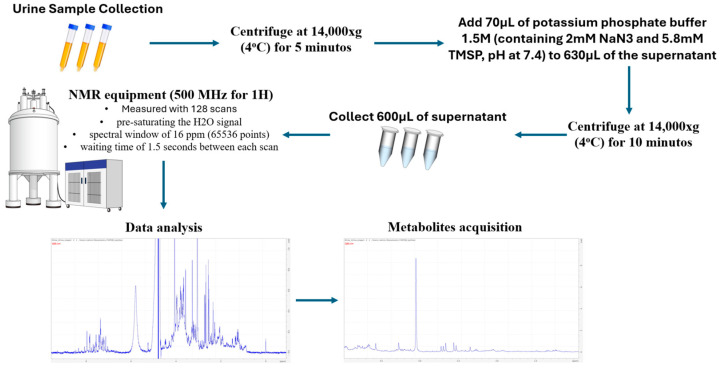
Experimental procedure carried out to determine the relevant urinary metabolites related to obesity.

A Python routine was developed to perform the spectral alignment of the signal of each metabolite of interest. Once the typical regions of the metabolite signals were aligned, these regions were integrated and used to assemble a new spreadsheet, where the data from the integrals were converted using the same routine into concentrations (mg/mL), using the concentration and signal of the TSP-4 standard at 0 ppm. Using these new data, bioinformatics analysis was carried out which filtered out the metabolite signals and selected those more relevant and related to obesity: 2-methylglutarate, 3-hydroxybutyrate, lactate, 2-hydroxyisobutyrate, alanine, acetate, nacetylglucosamine, lipids, citrate, pyruvate, carnitine, sarcosine, creatinine, acetylcholine choline, creatine, trigonelline, glucose, urea, phenylalanine, hippurate and formate. Figure 1 shows the main steps of the procedure.

### 2.6. Statistical Analysis

A descriptive analysis was conducted using frequency tables for categorical variables and position and dispersion measures for numerical variables. The Chi-square test or Fisher’s exact test were used to compare proportions when necessary. The Mann–Whitney test was used to compare numerical measurements between 2 groups and the Kruskal–Wallis test was used between 3 groups, followed by the Dunn test to locate differences, when necessary. Spearman’s linear correlation coefficient was used to assess the relationship between numerical variables. An ANOVA for repeated measures with rank transformation was used to compare the parameters between treatments and times. The computer program used was SAS System for Windows (Statistical Analysis System), version 9.4., SAS Institute Inc., 2002–2012, Cary, NC, USA. The ROC curve (Receiver Operating Characteristic curve) was used to assess the existence of a cutoff point that maximized the combination of sensitivity and specificity to predict visceral obesity. The computer program used was R version 4.2.0., copyright (C) 2022 The R Foundation for Statistical Computing. The significance level adopted for the statistical tests was 5% [42,43].

## 3. Results

### 3.1. Analysis of Body Composition, Demographic Data, Serum Biochemistry and Urinary Metabolites of Patients with Obesity or Overweight

Among the 75 patients, abdominal circumference varied between 82 and 93 cm in 15 patients, between 94 and 104 in 18 patients and >104 in 42 patients. Twenty-three patients had visceral fat levels between 9 and 16 kg and 52 had visceral fat >16 kg. In addition, 10 patients had a fat percentage <36%, 39 patients between 36 and 46%, and 26 patients >46% (Supplementary Material).

In terms of anthropometric parameters, the average visceral fat was 17.52 kg, waist circumference 108 cm, hips 117 cm, muscle mass 29 kg, fat mass 41 kg, height 157 cm, weight 93 kg, BMI 34.81 kg/m^2^, fat percentage 43.47%, waist-to-hip ratio 1.0 and basal metabolic rate 1506.31 kcal (Supplementary Material).

As for the biochemical analyses, the average fasting glucose was 86.32 mg/dL, glycated hemoglobin 5.53%, total cholesterol 184.04 mg/dL, HDL cholesterol 48.27 mg/dL, LDL cholesterol 104.43 mg/dL, triglycerides 146.40 mg/dL and uric acid 5.39 mg/dL (Supplementary Material).

### 3.2. Analysis of BMI Categories in Relation to Anthropometric, Biochemical, Demographic and Urinary Metabolite Parameters of Patients with Obesity or Overweight

The comparison between the three BMI groups (25–30, 30–35 and 35) showed an association between BMI ≥ 35 and glycated Hb (5.36 ± 0.50 vs. 5.63 ± 1.28 vs. 5.55 ± 0.41; *p* = 0.0430), uric acid (5.25 ± 1.78 vs. 4.96 ± 1.46 vs. 5.85 ± 1.29; *p* = 0.0350) and LDL cholesterol (97.63 ± 30.89 vs. 99.00 ± 26.69 vs. 113.43 ± 30.19; *p* = 0.0188). Higher resistance exercise time per week was associated with a BMI of 30–35 kg/m^2^ (17.37 ± 52.26 vs. 67.31 ± 106.72 vs. 14.00 ± 43.68; *p* = 0.0300). Age, sex, blood glucose, total and HDL cholesterol, triglycerides, aerobic exercise time and alcohol intake showed no difference among the three BMI groups (Supplementary Materials). There was no difference between the BMI groups in terms of the urinary metabolites studied (Table 1).

### 3.3. Analysis of Abdominal Circumference Categories in Relation to Anthropometric, Biochemical, Demographic and Urinary Metabolic Parameters

In the abdominal circumference categories (81–93, 94–104 and >104 cm), there was an association between higher uric acid concentrations and abdominal circumference ≥104 cm (4.66 ± 1.84 vs. 4.87 ± 1.15 vs. 5.87 ± 1.38, *p* = 0.0022). Females had higher frequencies of abdominal circumferences between 81 and 93 cm, and 94 and 104 cm, than males (100.0% vs. 0.0% and 83.3% vs. 16.7%; *p* = 0.0031), with similar frequencies for measurements ≥104 cm. There was no difference between the abdominal circumference categories and other anthropometric, biochemical and demographic parameters (Supplementary Materials). There was no difference between the abdominal circumference categories in terms of the urinary metabolites studied (Table 2).

### 3.4. Analysis of Visceral Fat Categories in Relation to Anthropometric, Biochemical, Demographic and Urinary Metabolic Parameters

Visceral fat ≥ 16 kg was associated with higher concentrations of LDL cholesterol (94.22 ± 33.01 vs. 108.94 ± 27.32; *p* = 0.0194). There was no difference between the visceral fat categories and other anthropometric, biochemical and demographic parameters (Supplementary Materials). As for the metabolomic profile, visceral fat ≥ 16 kg was associated with higher concentrations of trigonelline, sarcosine and phenylalanine (Table 3).

### 3.5. Analysis of Fat Percentile Categories in Relation to Demographic, Anthropometric, Biochemical and Urinary Metabolic Parameters

As for the fat percentile category (≤36%, 36–46% and ≥46%), higher uric acid concentrations were associated with a fat percentile ≤ 36% (6.38 ± 1.72 vs. 5.32 ± 1.52 vs. 5.11 ± 1.32; *p* = 0.0426). Female sex was associated with fat ≥46% and male sex was associated with fat ≤36% (96.2% vs. 3.8% and 80% vs. 20%; *p* < 0.0001, respectively). There was no difference between the fat percentile categories and other anthropometric, biochemical and demographic parameters (Supplementary Materials). There was no difference between the fat percentile categories in terms of urinary metabolomic profile (Table 4).

### 3.6. Discrimination of Visceral Fat from 9 to 16 kg or ≥16 kg by Trigonelline, Sarcosine and Phenylalanine Urinary Concentrations (ROC Curve)

**Figure 2:**
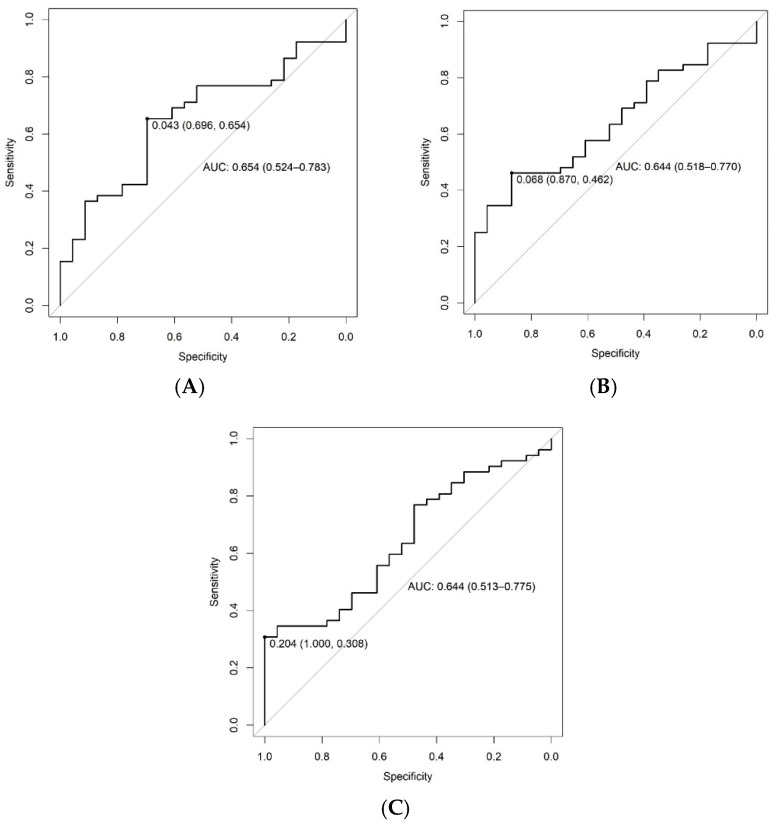
ROC curve representing urinary concentrations of the urinary metabolites sarcosine (**A**), trigonelline (**B**) and phenylalanine (**C**) for the discrimination of patients presenting amounts of visceral fat from 9 to 16 kg or ≥16 kg.

A ROC curve was constructed for each of the metabolites trigonelline, sarcosine and phenylalanine in order to determine a cutoff point in urinary concentrations to discriminate patients with visceral fat from 9 to 16 kg or ≥16 kg. Table 5 and Figure 2 demonstrate the accuracy of metabolites in predicting visceral obesity.

## 4. Discussion

This study evaluated the urinary metabolomic profiles of patients with obesity or overweight according to BMI, abdominal circumference, percentage of body fat and amount of visceral fat, parameters that represent important characteristics of these patients.

Metabolites related to the pathophysiological mechanisms of obesity have been identified, but interestingly, there was only an association between the urinary concentrations of trigonelline, sarcosine and phenylalanine and a greater amount of visceral fat being observed by bioimpedance. Trigonelline, sarcosine and phenylalanine play significant roles in regulating energy balance and the metabolic pathways essential for controlling obesity. It is known that visceral adiposity reflects the accumulation of fat around the internal organs and is related to various metabolic and metabolomic disorders. Some authors have found that excess visceral fat, even when assessed by bioimpedance, is associated with insulin resistance, chronic low-grade inflammation and increased risk of developing type 2 diabetes and cardiovascular disease [44].

Phenylalanine, an essential amino acid obtained from food, is converted into tyrosine by phenylalanine hydroxylase, influencing metabolic pathways as gluconeogenesis. The presence of phenylalanine in certain foods and artificial sweeteners, such as aspartame, highlights its importance. It can be metabolized to fumarate and acetoacetate, suggesting an indirect role in gluconeogenesis, and studies have shown that acetate can regulate a variety of cytokines in adipose tissue, inhibit the oxidative decomposition of fatty acids in the liver and thus increase lipid deposition. These highlights show the potential impact of phenylalanine and acetate on visceral fat and the development of hepatic steatosis [45,46,47,48].

Tanaka et al. [49] found that people with a large amount of abdominal fat had higher concentrations of phenylalanine in their blood compared to those with less visceral fat. Schlecht et al. [50] observed that in men, visceral fat was associated with higher blood levels of essential amino acids such as valine, isoleucine and phenylalanine. It is also known that patients with classic phenylketonuria have high concentrations of phenylalanine and can gain weight more easily than those without the condition, and have larger waists and unhealthy lipogenesis and amounts of body fat. It is believed that in such patients, due to their restrictive diet, there is a higher intake of carbohydrates and lower energy expenditure in moderate physical activity, which can contribute to obesity [51]. These studies have shown a possible direct relationship between the presence of fat in different regions, such as the abdomen, and high concentrations of phenylalanine. In part, these findings corroborate the data obtained in the present study, which showed that there were greater amounts of visceral fat in patients who had higher concentrations of phenylalanine in their urine. The relevance of this relationship lies in the possibility of phenylalanine being used in the future as a possible biomarker for metabolic diseases.

Another metabolite that stood out among patients with a higher degree of visceral obesity was trigonelline. It is a precursor of nicotinamide adenine dinucleotide (NAD+). Naturally synthesized by various plant species, it is produced by the intestinal microbiome in humans. Found in high concentrations in coffee and its by-products, serum trigonelline levels are independent of the dietary intake of caffeine and vitamin B3. Studies suggest that trigonelline may act as an NAD+ precursor, potentially influencing mitochondrial function and muscle health. Individuals with sarcopenia have lower serum trigonelline levels, which correlate directly with muscle mass, handgrip strength and gait speed, important parameters used in the diagnosis of sarcopenia [52,53,54]. Mitochondrial oxidative phosphorylation, a crucial process for energy production in cells, has been shown to be associated with trigonelline levels [53]. Trigonelline also stimulates the p38 MAPK/ATF-2 signaling pathway, leading to the activation of proteins and genes characteristic of brown fat, as well as genes specific to beige and white adipose tissue. The activation of these pathways increases lipid metabolism in white adipocytes by decreasing adipogenesis and lipogenesis, promoting lipolysis and increasing fatty acid oxidation.

Several researchers have investigated the impact of trigonelline on the regulation of hepatocellular autophagy, especially under high-fat diet conditions. The dysfunction of hepatic autophagy is frequently associated with nonalcoholic fatty liver disease (NAFLD), often present in patients with increased visceral fat, and the potential of trigonelline in restoring this process may play a crucial role in its hepatoprotective properties [55,56]. Furthermore, recent studies have demonstrated the role of trigonelline as an excellent antidiabetic agent through increasing the activity of carnitine, palmitoyl transferase and glucokinase, being able to improve glucose uptake and insulin resistance [57].

The third metabolite that was elevated in patients with visceral obesity was sarcosine. This metabolite is found in the kidney and liver, and could potentially serve as an early marker of metabolic syndrome in individuals with obesity, justifying investigation.

In insulin-resistant individuals, the levels of sarcosine, an intermediate compound in the synthesis and degradation of glycine, are high. This increase can be attributed to changes in the excretion of this metabolite in the urine, indicating a change in the glycine metabolism pathways in these patients. Glycine metabolism is intrinsically linked to that of branched-chain amino acids (BCAAs), as it plays a crucial role as a donor in the pyruvate/alanine cycle. In conditions such as obesity and diabetes, where there is an increase in BCAA levels, there is a corresponding decrease in glycine levels due to the interaction between these metabolic pathways. High BCAA levels have been implicated in the activation of the mammalian target of rapamycin (mTOR) signaling pathway, leading to oxidative stress, mitochondrial dysfunction and cell apoptosis, mechanisms that potentially contribute to the pathophysiology of obesity and related diseases [58,59]. There therefore seems to be a direct biological connection between higher sarcosine concentrations and higher visceral obesity, which was also demonstrated in our study. It is important to highlight that, although urinary sarcosine has already been investigated in previous studies as a possible marker of diabetes, its use as an indicator of visceral obesity is something unprecedented [60].

In order to enable the discrimination of patients with visceral fat from 9 to 16 kg or ≥16 kg, a ROC curve was constructed for each of the metabolites trigonelline, sarcosine and phenylalanine. It was possible to determine a cutoff point in the urinary concentration that discriminated the amount of visceral fat with good accuracy and positive predictive values. These cutoff points may be useful for the possible estimation of metabolites associated with visceral fat, known to be closely related to increased cardiovascular risk. Visceral fat releases inflammatory interleukins such as tumor necrosis factor alpha (TNF-α) and interleukin 6, which impair insulin action in the liver, leading to increased gluconeogenesis and the synthesis of triglyceride-rich very-low-density lipoprotein (VLDL) particles and contributing to metabolic disorders associated with visceral fat [61,62,63]. In addition, urinary dosages appear as an interesting option for non-invasive estimation of these and other metabolites.

When we looked at the other categories related to obesity, such as body fat percentage, abdominal circumference and BMI, and compared them to the findings of the metabolomic profile, there was no identification of metabolites specifically related to these characteristics. This finding is therefore important, as these parameters do not allow us to distinguish between subcutaneous and visceral adipose tissue, resulting in less specific characterization for differentiating metabolic pathways. We emphasize that even using a method not considered the gold standard for assessing body composition, bioimpedance was able to identify patients with a higher visceral fat content and relate it to important metabolites associated with the pathophysiological process of obesity.

We found an association between serum uric acid and BMI. Similar results were found in the literature by Li et al. [64] and Dalbeth et al. [65], where they found that patients with higher BMI had higher de novo purine synthesis or lower renal clearance of uric acid.

In addition, there was an association between BMI and LDL cholesterol and glycated Hb. This has been previously shown in other studies, but there is no consensus in the literature, especially regarding LDL cholesterol. Particularly in patients with diabetes and a higher BMI, there is an association with higher levels of LDL cholesterol. A higher BMI would increase the expression of genes involved in lipid metabolism, leading to greater synthesis of LDL cholesterol and reducing its elimination. As for glycated Hb, the association with higher BMI is well established and reinforces the importance of maintaining adequate body weight for the prevention of type 2 diabetes mellitus [66,67,68,69].

It was found that patients with greater abdominal circumference had higher concentrations of LDL cholesterol and uric acid. This relationship indicates a potential impact of abdominal adiposity on uric acid and lipid metabolism in the population studied, in agreement with the literature [70,71].

Fat percentile was not a good predictor of metabolic alterations in the population studied; however, visceral fat was associated with higher levels of LDL cholesterol. Some authors [72,73] highlight the importance of considering regional visceral adiposity when assessing cardiovascular risk factors, since visceral adipose tissue is related to subclinical chronic inflammation, which can lead to endothelial dysfunction, insulin resistance and an increased risk of cardiometabolic diseases.

It is important to highlight that phenylalanine is an indirect intermediate of the tricarboxylic acid cycle and is involved in gluconeogenesis, which justifies the relationship with visceral fat found in this study. Trigonelline demonstrates a potential effect in attenuating the impacts of visceral fat. The increase in its urinary excretion can, therefore, be attributed to an increase in intestinal absorption in patients with greater visceral fat, possibly as a mechanism of metabolic self-regulation. In turn, the increase in urinary sarcosine may be a direct reflection of the link between visceral fat and insulin resistance, where high levels of sarcosine may be a consequence of changes in the metabolism of glycine, which is interconnected with the metabolism of BCAAs, very present in these patient profiles. Studies showed that these three metabolites are also associated with a greater chance of developing other diseases [74].

The strengths of this study include its prospective nature and the evaluation of the urinary metabolomic profile with a focus on different types of obesity and overweight, which may represent a more accessible way of evaluating metabolites than collecting peripheral blood. Among the limiting factors, urinary metabolomics is complex and unusual, which makes it difficult to analyze and obtain data for the acquisition of metabolites. The targeted approach to some metabolites was another limitation of this study as it may have neglected other metabolites that were related to metabolic alterations in adipose tissue. Extending this study with a greater number of metabolites to include other metabolic pathways is necessary. In addition, this study did not integrate data from transcriptomic (gene expression) and proteomic (protein expression) analyses, useful approaches for providing complementary information on the molecular processes occurring in adipose tissue.

## 5. Conclusions

In conclusion, higher visceral fat was associated with urinary levels of metabolites such as sarcosine, related to insulin resistance; trigonelline, related to muscle mass and strength; and phenylalanine, related to glucose metabolism and abdominal fat. Trigonelline, sarcosine and phenylalanine play significant roles in regulating energy balance and metabolic pathways essential for controlling obesity. Possible alterations in specific metabolic pathways, such as gluconeogenesis, lipid metabolism, glucose uptake, insulin resistance and the metabolism of other amino acids such as glycine and BCAA may occur in the presence of higher concentrations of these metabolites in obese and overweight patients with larger amounts of visceral fat. Our findings could represent an interesting option for the non-invasive estimation of visceral fat through biomarkers related to alterations in metabolic pathways involved in the pathophysiology of obesity. However, further studies will be needed to corroborate the present findings.

## Figures and Tables

**Table 1:** Comparisons between BMI categories and the urinary metabolites studied.

Variable	BMI 25–30 kg/m^2^ (*n* = 19)	BMI 30–35 kg/m^2^ (*n* = 26)	BMI ≥ 35 kg/m^2^ (*n* = 30)	*p*-Value
Valine	0.00061546 ± 0.00505177	0.00538768 ± 0.01269377	0.00014959 ± 0.01684119	0.1033
2-Methylglutarate	0.00165881 ± 0.01655289	0.00538768 ± 0.01269377	0.00014959± 0.01684119	0.1179
PropyleneGlycol	0.00090099 ± 0.03005212	0.01640011 ± 0.03826574	−0.00205988 ± 0.05979712	0.1313
3-Hydroxybutyrate	0.02010601 ± 0.06175205	0.08954448 ± 0.32246036	−0.00302254 ± 0.15196923	0.4294
Methylmalonate	0.01264350 ± 0.11264410	0.14365927 ± 0.36298013	−0.00404615 ± 0.40640139	0.0891
3-Hydroxyisovalerate	0.00390361 ± 0.02444776	0.02586556 ± 0.05433844	−0.00296892 ± 0.08594212	0.1105
Lactate	0.00937613 ± 0.06128961	0.06418234± 0.17184152	−0.01809144 ± 0.23604109	0.0963
2-Hydroxyisobutyrate	0.00281533 ± 0.01369690	0.01522823 ± 0.03859131	−0.00273186 ± 0.05487979	0.0998
Alanine	0.01237974 ± 0.06928748	0.06809089 ± 0.18076744	−0.00930114 ± 0.25718270	0.1789
Acetate	0.00437516 ± 0.03796163	0.04344184 ± 0.09805174	−0.01301485 ± 0.15391431	0.1407
Nacetylglucosamine	0.02468230 ± 0.11157969	0.11868568 ± 0.26663178	−0.03213317 ± 0.51034783	0.1360
Lipids	0.07097576 ± 0.32677337	0.35051343 ± 0.77455655	−0.09941926 ± 1.51964602	0.1204
Citrate	0.03605499 ± 0.30676713	0.30029714 ± 0.59024590	0.02023172 ± 0.93709287	0.2556
Piruvate	0.00552071 ± 0.16038939	0.13064686 ± 0.28647585	−0.07837194 ± 0.55049093	0.2342
Carnitine	0.00218021 ± 0.02079422	0.02055738 ± 0.04867125	−0.00508360 ± 0.07319585	0.2123
Sarcosine	0.02019644 ± 0.09732506	0.12127916 ± 0.30305130	−0.03881738 ± 0.47332956	0.1137
Creatinine	0.38869472 ± 3.01703968	3.25117597 ± 6.96024323	0.38856177 ± 10.0466937	0.1344
AcetylcholineCholine	0.00455496 ± 0.02257989	0.02897809 ± 0.07532024	−0.00892429 ± 0.11170803	0.1127
Methanol	0.00361544 ± 0.01424125	0.02033866 ± 0.05404970	−0.00490569 ± 0.07154278	0.3840
Creatine	0.10509202 ± 0.45258149	0.63520803 ± 1.25169756	0.00504035 ± 1.68803007	0.1385
Lactate	0.01789560 ± 0.09689388	0.11237576 ± 0.30115361	−0.02013265 ± 0.38551300	0.1893
Trigonelline	0.02536680 ± 0.09299115	0.13292601 ± 0.36331211	−0.00443190 ± 0.50497876	0.1871
Glucose	0.01127453 ± 0.03706481	0.11815990 ± 0.41238564	0.05508906 ± 0.42044034	0.3838
Urea	0.33765854 ± 1.62787013	2.19163044 ± 6.64806586	−0.63307152 ± 6.84119576	0.4810
Phenylalanine	0.02472187 ± 0.38385305	0.27918801 ± 0.59437347	−0.19283182 ± 1.16757047	0.2072
Hippurate	0.16583012 ± 0.33340075	0.72081954 ± 1.75203825	−0.27248268 ± 2.54214070	0.4637
Formate	0.00566661 ± 0.01631842	0.01752892 ± 0.03717082	−0.00544484 ± 0.08135147	0.3593

Values represented as mean ± standard deviation.

**Table 2:** Comparison between patients with obesity or overweight according to abdominal circumference and the urinary metabolites studied.

Variable	Abdominal Circumference			*p*-Value
	81–93 cm (*n* = 15)	94–104 cm (*n* = 18)	≥104 cm (*n* = 42)	
Valine	−0.00003918 ± 0.0059266	0.00640189 ± 0.0153453	0.00099083 ± 0.0141434	0.1816
2-Methylglutarate	−0.00099108 ± 0.0207314	0.01975488 ± 0.0455552	0.00131910 ± 0.0503638	0.2003
PropyleneGlycol	−0.00110667 ± 0.0204604	0.02010409 ± 0.0607330	0.00517802 ± 0.05418077	0.0740
3-Hydroxybutyrate	0.00370326 ± 0.11563711	0.11983077 ± 0.3755580	0.00969027 ± 0.13129364	0.7392
Methylmalonate	0.00037540 ± 0.13141282	0.17016515 ± 0.4431974	0.01869951 ± 0.33984214	0.1442
3-Hydroxyisovalerate	−0.00005216 ± 0.02897823	0.02996702 ± 0.0654515	0.00283289 ± 0.07252166	0.1495
Lactate	−0.00863083 ± 0.10494395	0.07997944 ± 0.1903446	−0.00014341 ± 0.20084090	0.2650
2-Hydroxyisobutyrate	−0.00016439 ± 0.02021745	0.01935372 ± 0.0456693	0.00051353 ± 0.04591141	0.2219
Alanine	−0.00506805 ± 0.10705881	0.09296386 ± 0.2108559	0.00307655 ± 0.21384420	0.1934
Acetate	0.00092327 ± 0.04023385	0.04615098 ± 0.1121220	−0.00053325 ± 0.13371478	0.1854
Nacetylglucosamine	0.00092327 ± 0.04023385	0.14632220 ± 0.3265442	−0.00476474 ± 0.42717293	0.3087
Lipids	0.03263438 ± 0.40939744	0.42623953 ± 0.9511242	−0.01625039 ± 1.27312614	0.3189
Citrate	−0.01137573 ± 0.36231502	0.35305569 ± 0.6987732	0.06941323 ± 0.79118337	0.1543
Piruvate	−0.00053166 ± 0.15408531	0.13135252 ± 0.3456552	−0.02870993 ± 0.47428649	0.2558
Carnitine	0.00029096 ± 0.02298804	0.02438315 ± 0.0592505	−0.00047270 ± 0.06177039	0.3534
Sarcosine	0.00464012 ± 0.13274367	0.15163795 ± 0.3620950	−0.01015752 ± 0.39831673	0.2057
Creatinine	0.07774829 ± 3.44967844	4.23075194 ± 9.0271122	0.62506830 ± 8.10561314	0.2485
AcetylcholineCholine	−0.00027200 ± 0.03279491	0.03616655 ± 0.0889678	−0.00177790 ± 0.09425045	0.2140
Methanol	0.00157884 ± 0.01582618	0.02694244 ± 0.0662672	−0.00138854 ± 0.05957149	0.3289
Creatine	0.07819098 ± 0.51246949	0.78868285 ± 1.4908866	0.07843361 ± 1.42436138	0.1827
Lactate	0.00264651 ± 0.12198857	0.14613636 ± 0.3651196	−0.00029394 ± 0.32134385	0.1617
Trigonelline	0.00700087 ± 0.12808419	0.18859049 ± 0.4574680	0.00727254 ± 0.41000676	0.1667
Glucose	−0.00235783 ± 0.05865739	0.17549902 ± 0.4900429	0.04322453 ± 0.35253559	0.2527
Urea	−0.08895897 ± 2.38083542	2.84288549 ± 7.7893955	−0.1293284 ± 5.82640239	0.2457
Phenylalanine	0.05580632 ± 0.30766599	0.26810350 ± 0.7645377	−0.08855496 ± 1.00291494	0.1296
Hippurate	0.09461949 ± 0.36748316	0.84006634 ± 2.0311798	−0.06721158 ± 2.19399656	0.2275
Formate	0.00308861 ± 0.01812276	0.02268495 ± 0.0447608	−0.00129967 ± 0.06854088	0.2712

Values represented as mean ± standard deviation.

**Table 3:** Comparisons between patients with obesity or overweight according to the amount of visceral fat and the urinary metabolites studied.

Variable	Visceral Fat 9–16 kg *n* = 23	Visceral Fat ≥ 16 kg *n* = 52	*p*-Value
Valine	0.00079868 ± 0.00485752	0.00265176 ± 0.01570310	0.0858
2-Methylglutarate	0.00219207 ± 0.01581101	0.00664819 ± 0.05322867	0.0557
PropyleneGlycol	0.00110070 ± 0.02794615	0.01033527 ± 0.05850736	0.0858
3-Hydroxybutyrate	0.01715363 ± 0.05389605	0.04278771 ± 0.25776019	0.1697
Methylmalonate	0.02620161 ± 0.13164189	0.06252589 ± 0.40224233	0.0817
3-Hydroxyisovalerate	0.00521253 ± 0.02460903	0.01034072 ± 0.07633910	0.1922
Lactate	0.01016681 ± 0.05763499	0.01034072 ± 0.07633910	0.1222
2-Hydroxyisobutyrate	0.00309513 ± 0.01308270	0.00569772 ± 0.05023104	0.1115
Alanine	0.01072919 ± 0.06342359	0.02845716 ± 0.23490839	0.1065
Acetate	0.00402614 ± 0.03489568	0.01403017 ± 0.13794045	0.0572
Nacetylglucosamine	0.02092272 ± 0.10046501	0.04056872 ± 0.43489233	0.0759
Lipids	0.06373849 ± 0.29764104	0.11564087 ± 1.28901603	0.0740
Citrate	0.04883437 ± 0.30544026	0.15339484 ± 0.82747618	0.1526
Piruvate	0.00095599 ± 0.14409099	0.02170318 ± 0.47383020	0.1090
Carnitine	0.00245187 ± 0.01990206	0.00705797 ± 0.06609374	0.1251
Sarcosine	0.01791004 ± 0.09199581	0.03770266 ± 0.42314367	0.0350
Creatinine	0.73891369 ± 3.26887661	1.66495410 ± 9.06680192	0.3092
AcetylcholineCholine	0.00519861 ± 0.02148845	0.00870534 ± 0.10124550	0.0923
Methanol	0.00323570 ± 0.01373972	0.00722897 ± 0.06711590	0.1733
Creatine	0.13589880 ± 0.50873213	0.29880183 ± 1.56824870	0.1308
Trigonelline	0.01992715 ± 0.08161980	0.06436085 ± 0.46340115	0.0488
Glucose	0.00899314 ± 0.03565325	0.09100390 ± 0.42928575	0.4245
Urea	0.35799561 ± 1.56831964	0.69561268 ± 7.09422371	0.1922
Phenylalanine	−0.00766327 ± 0.30258184	0.04076740 ± 1.01042900	0.0488
Hippurate	0.12361041 ± 0.31564652	0.20912617 ± 2.33303284	0.1222
Formate	0.00532253 ± 0.01497627	0.00533950 ± 0.06768999	0.3038

Values represented as mean ± standard deviation.

**Table 4:** Descriptive analysis and comparisons between groups according to total fat percentile and metabolomic comparison between different fat percentile groups.

Variable	Total Fat Percentile			*p*-Value
	≤36% (*n* = 10)	36–46% (*n* = 39)	≥46% (*n* = 26)	
Valine	0.00101265 ± 0.00512192	0.00450994 ± 0.01139156	−0.00114435 ± 0.01728648	0.7945
2-Methylglutarate	0.00314260 ± 0.01611409	0.01467480 ± 0.03594132	−0.00798537 ± 0.06027488	0.8306
PropyleneGlycol	0.00409456 ± 0.01609466	0.01783636 ± 0.05067407	−0.00668513 ± 0.05786045	0.8664
Ethano1	0.00000032 ± 0.00000065	0.00000290 ± 0.00001346	0.00000711 ± 0.00002287	0.5312
3-Hydroxybutyrate	0.02643885 ± 0.06423856	0.06514708 ± 0.26499481	−0.00713964 ± 0.16430558	0.6539
Methylmalonate	0.04090139 ± 0.16637206	0.12306473 ± 0.32330034	−0.05209827 ± 0.39764965	0.8468
3-Hydroxyisovalerate	0.00617340 ± 0.02663063	0.02253102 ± 0.05056713	−0.01087839 ± 0.08715801	0.9368
Lactate	0.01293351± 0.05698275	0.05047227 ± 0.14923777	−0.03052344 ± 0.24814429	0.8900
2-Hydroxyisobutyrate	0.00348871 ± 0.01485912	0.01355455 ± 0.03461765	−0.00754019 ± 0.05587989	0.9319
Alanine	0.01249510 ± 0.06293919	0.06211563 ± 0.16490216	−0.03157372 ± 0.26081422	0.9454
Acetate	0.00491930 ± 0.03334036	0.03467889 ± 0.08651634	−0.02228845 ± 0.16165477	0.6730
Nacetylglucosamine	0.02486789 ± 0.10438933	0.11057482 ± 0.25199513	−0.07578080 ± 0.52229981	0.8673
Lipids	0.07362487 ± 0.30757253	0.32482659 ± 0.73261361	−0.22789135 ± 1.55834702	0.8430
Citrate	0.06499638 ± 0.32964008	0.25071499 ± 0.56684142	−0.05108179 ± 0.94872898	0.6722
Piruvate	0.00393686 ± 0.15424157	0.08876121 ± 0.25078126	−0.09040393 ± 0.59355393	0.5918
Carnitine	0.00364686 ± 0.02018337	0.01692661 ± 0.04392245	−0.01050764 ± 0.07565114	0.7559
Citrate	0.06745977 ± 0.33477574	0.24284605 ± 0.55647571	−0.02256917 ± 0.90172737	0.7119
Sarcosine	0.02231522 ± 0.10276815	0.10630231 ± 0.26981221	−0.07678743 ± 0.48738792	0.8166
Creatinine	0.60808788 ± 2.88463903	3.17411222 ± 7.16106132	−1.01148568 ± 9.24674203	0.8177
AcetylcholineCholine	0.00565693 ± 0.02326015	0.02494170 ± 0.06599811	−0.01757883 ± 0.11567202	0.8204
Methanol	0.00350903 ± 0.01478470	0.01822024 ± 0.04783386	−0.01135970 ± 0.07252683	0.7426
Creatine	0.08155832 ± 0.30586861	0.44641898 ± 1.02869271	0.01682478 ± 1.86859026	0.7239
Lactate	0.01928254 ± 0.08798780	0.09675085 ± 0.26694490	−0.05031931 ± 0.39399175	0.7291
Trigonelline	0.02374965 ± 0.08399346	0.13526169 ± 0.34584082	−0.06567746 ± 0.48509884	0.7405
Glucose	0.00491625 ± 0.04189189	0.03958419 ± 0.09995621	0.12869613 ± 0.59905361	0.5960
Urea	0.25569148 ± 1.79448506	1.81532837 ± 5.54362472	−1.11342165 ± 7.18541052	0.6830
Phenylalanine	0.00579840 ± 0.23872775	0.16557466 ± 0.52906930	−0.17583641 ± 1.28293793	0.3153
Hippurate	0.12922105 ± 0.22979078	0.53681284 ± 1.45850899	−0.32731966 ± 2.73430691	0.5870
Formate	0.00496132 ± 0.01400679	0.01507905 ± 0.03273125	−0.00913938 ± 0.08648353	0.8986

Values represented as mean ± standard deviation or number of patients and percentile.

**Table 5:** Accuracy of sarcosine, trigonelline and phenylalanine in predicting visceral obesity.

Metabolites	Accuracy AUC	CL_AUC_	Cutoff Point	Sensibility	Specificity	Positive Predictive Value	Negative Predictive Value
Sarcosine (mg/mL)	0.654	0.524; 0.783	0.043	65%	69%	83%	47%
Trigonelline (mg/mL)	0.644	0.518; 0.770	0.068	46%	87%	89%	42%
Phenylalanine (mg/mL)	0.644	0.513; 0.775	0.204	31%	100%	100%	39%

CL_AUC_ = Confidence limits of the area under the curve.

## Data Availability

The original contributions presented in the study are included in the article/supplementary material, further inquiries can be directed to the corresponding author.

## Supplementary Materials

The following supporting information can be downloaded at: https://www.mdpi.com/article/10.3390/metabo14090491/s1, Table S1: Demographic parameters, body composition and metabolic changes in patients with obesity or overweight, Table S2: Anthropometric and body composition parameters obtained by bioimpedance and anthropometry of patients with obesity or overweight.; Table S3: Biochemical parameters of glucose, lipid and uric acid metabolism in patients with obesity or overweight; Table S4: Comparison between patients with obesity or overweight according to BMI categories and demographic, lifestyle and biochemical parameters; Table S5: Comparison between patients with obesity or overweight according to abdominal circumference and demographic, lifestyle and biochemical parameters.; Table S6: Comparisons between patients with obesity or overweight according to the amount of visceral fat and demographic, lifestyle and biochemical parameters. Table S7. Comparisons between patients with obesity or overweight according to the total fat percentile and demographic, lifestyle and biochemical parameters.
