# Supplementary material for: Sarcosine, Trigonelline and Phenylalanine as Urinary Metabolites Related to Visceral Fat in Overweight and Obesity
Source: Metabolites. 2024 Sep 10;14(9):491. doi: 10.3390/metabo14090491 (PMC11434364; doi:10.3390/metabo14090491)
Supplement: Supplementary file 1 [file metabolites-14-00491-s001.zip › metabolites-3153693-supplementary.pdf]

metabolites

MDPI

Supplementary Material

Table S1. Demographic parameters, body composition and metabolic changes in patients with obesity or overweight.

VARIABLE		PATIENTS (n = 75)
Age (years)		38.33 ± 8.55
Sex	Female	54 (72%)
	Male	21 (28%)
BMI (Kg/m²)	25-30	19 (25.3%)
	30-35	26 (34.7%)
	≥35	30 (40.0%)
Abdominal Circumference (cm)	82-93	15 (20%)
	94-104	18 (24.0%)
	>104	42 (56.0%)
Visceral Fat (Kg)	<16	23 (30.7%)
	>16	52 (69.3%)
Percentile Body Fat (%)	<36%	10 (13.3%)
	36%-46%	39 (52.0%)
	>46%	26 (34.7%)

Categorical variables are represented as frequency (n = absolute number and % percentage). Continuous variables are represented as mean ± standard deviation.

Table S2. Anthropometric and body composition parameters obtained by bioimpedance and anthropometry of patients with obesity or overweight.

Variable	Mean ± SD	Median [Min;Max]
Visceral fat (kg)	17.52 ± 3.15	19.00 (9.00-20.00)
Abdominal waist circumference (cm)	108.28 ± 15.74	106.00 (81.00-158.00)
Hip (cm)	118.66 ± 13.31	117.00 (97.00-164.00)
Skeletal muscle mass (Kg)	29.38 ± 7.08	27.50 (18.90-48.40
Crude fat mass (Kg)	41.18 ± 12.92	38.10 (20.90-84.00)
Height (cm)	157.00 (1.45-182.50)	109.22 ± 76.85
Weight (kg)	93.72 ± 20.28	87.70 (61.50-153.60)
Body mass index (kg/m²)	34.81 ± 6.39	32.40 (25.60-55.30)
Body fat percentile (%)	43.47 ± 7.02	43.80 (29.10-56.80)
Waist to hip ratio	1.00 ± 0.07	0.99 (0.88-1.40)
Basal metabolic rate (kcal)	1506.31 ± 251.74	1441.00 (1138.00-2180.00)

Values represented as mean±standard deviation and median standard deviation

Table S3. Biochemical parameters of glucose, lipid and uric acid metabolism in patients with obesity or overweight.

Variable	Mean ± SD	Median [Min;Max]
Glucose (mg/dL)	86.32 ± 22.35	84.00 (59.00-237.00)
HbA1c (%)	5.53 ± 0.83	5.31 (4.78-11.71)
Total cholesterol (mg/dL)	184.04 ± 37.60	184.00 (88.00-288.00)
HDL (mg/dL)	48.27 ± 10.13	48.00 (15.00-73.00)
LDL (mg/dL)	104.43 ± 29.75	107.00 (30.00-177.00)
Triglyceride (mg/dL)	146.40 ± 113.98	107.00 (51.00-676.00)
Uric Acid (mg/dL)	5.39 ± 1.52	5.30 (2.70-9.70)

Values represented as mean±standard deviation and median±standard deviation

Table S4. Comparison between patients with obesity or overweight according to BMI categories and demographic, lifestyle and biochemical parameters.

Variable		BMI 25-30 kg/m² (n=19)	BMI 30-35 kg/m² (n=26)	BMI ≥35 kg/m² (n=30)	p-value
Age (years)		38.42 ± 8.87	36.35 ± 8.69	40.00 ± 8.12	0.1981¹
Glucose (mg/dL)		84.37 ± 19.01	89.04 ± 31.71	85.17 ± 12.70	0.5596¹
HbA1c (%)		5.36 ± 0.50	5.63 ± 1.28	5.55 ± 0.41	0.0430¹#
Total cholesterol (mg/dL)		178.89 ± 33.87	181.23 ± 44.40	189.73 ± 33.73	0.2466¹
HDL (mg/dL)		48.74 ± 10.26	51.00 ± 9.94	45.60 ± 9.86	0.2252¹
LDL (mg/dL)		97.63 ± 30.89	99.00 ± 26.69	113.43 ± 30.19	0.0188¹#
Triglyceride (mg/dL)		153.26 ± 138.84	136.42 ± 125.57	150.70 ± 86.35	0.2580¹
Uric Acid (mg/dL)		5.25 ± 1.78	4.96 ± 1.46	5.85 ± 1.29	0.0350¹#
Resistance exercise time (min/week)		17.37 ± 52.26	67.31 ± 106.72	14.00 ± 43.68	0.0300¹#
Aerobic exercise time (min/week)		68.42 ± 85.65	99.23 ± 149.34	43.67 ± 90.84	0.2032¹
Sex	Female	13 (68.4%)	21 (80.8%)	20 (66.7%)	0.4640²
	Male	6 (31.6%)	5 (19.2%)	10 (33.3%)	
Alcohol intake	> 150 g/week	8 (42.1%)	10 (40.0%)	13 (43.3%)	0.9691²
	< 150 g/week	11 (57.9%	15 (60.0%)	17 (56.7%)	

Categorical variables are represented as frequency (n = absolute number and % percentage). Continuous variables are represented as mean ± standard deviation. 1= based on Kruskal-Wallis test; 2= based on Chi-square test #differences between (Dunn's test): 81-93 and >104; 94-104 and >104cm. Values represented as mean±standard deviation.

Table S5. Comparison between patients with obesity or overweight according to abdominal circumference and demographic, lifestyle and biochemical parameters.

Variable		Abdominal circumference			p-value
		81-93 cm (n=15)	94-104 cm (n=18)	≥ 104 cm (n=42)	
Age (Years)		34.20 ± 6.66	38.00 ± 8.55	39.95 ± 8.80	0.0978[1]
Glucose (mg/dL)		80.40 ± 8.95	90.06 ± 38.73	86.85 ± 15.04	0.1358[1]
HbA1c (%)		5.29 ± 0.26	5.72 ± 1.52	5.53 ± 0.48	0.1931[1]
Total cholesterol (mg/dL)		168.47 ± 27.42	182.50 ± 42.32	190.26 ± 37.72	0.1340[1]
HDL (mg/dL)		54.07 ± 9.77	46.72 ± 10.93	46.86 ± 9.36	0.0656[1]
LDL (mg/dL)		91.80 ± 25.52	102.39 ± 32.05	109.81 ± 29.31	0.0559[1]
Triglyceride (mg/dL)		100.53 ± 35.70	167.33 ± 145.07	153.81 ± 115.19	0.1496[1]
Uric acid (mg/dL)		4.66 ± 1.84	4.87 ± 1.15	5.87 ± 1.38	0.0022[1#]
Weekly resistance exercise time (min)		22.00 ± 58.33	41.11 ± 70.78	34.05 ± 85.46	0.5831[1]
Weekly aerobic exercise time (min)		65.33 ± 88.55	71.67 ± 93.26	69.52 ± 131.96	0.7538[1]
Sex	Female	15 (100.0%)	15 (83.3%)	24 (57.1%)	0.0031[2]
	Male	0 (0.0%)	3 (16.7%)	18 (42.9%)	
Alcohol intake	> 150 g/week	6 (40.0%)	7 (41.2%)	18 (42.9%)	0.9794[2]
	< 150 g/week	9 (60.0%)	10 (58.8%)	24 (57.1%)	

Categorical variables are represented as frequency (n = absolute number and % percentage). Continuous variables are represented as mean ± standard deviation. 1= based on Kruskal-Wallis test; 2= based on Chi-square test #differences between (Dunn's test): 81-93 and >104; 94-104 and >104cm. Values represented as mean±standard deviation.

Table S6. Comparisons between patients with obesity or overweight to the amount of visceral according fat and demographic, lifestyle and biochemical parameters.

Variable		Visceral fat 9-16 kg n=23	Visceral fat ≥16 kg n=52	p-value
Age (Years)		37.57 ± 8.88	38.67 ± 8.46	0.5927[1]
Glucose (mg/dL)		85.48 ± 18.28	86.71 ± 24.12	0.7881[1]
HbA1c (%)		5.42 ± 0.46	5.58 ± 0.95	0.5014[1]
Total cholesterol (mg/dL)		181.52 ± 48.02	185.15 ± 32.44	0.5236[1]
HDL (mg/dL)		46.26 ± 9.99	49.15 ± 10.16	0.1747[1]
LDL (mg/dL)		94.22 ± 33.01	108.94 ± 27.32	0.0194[1]
Triglyceride (mg/dL)		174.65 ± 170.63	133.90 ± 75.98	0.8451[1]
Uric acid (mg/dL)		5.32 ± 1.50	5.42 ± 1.54	0.9771[1]
Weekly resistance exercise (min)		26.96 ± 77.60	36.15 ± 76.88	0.4466[1]
Weekly aerobic exercise (min)		78.70 ± 117.64	65.00 ± 114.15	0.4415[1]
Sex	Female	14 (60.9%)	40 (76.9%)	0.1534[2]
	Male	9 (39.1%)	12 (23.1%)	
Alcohol intake	≥150 g/week	8 (34.8%)	23 (45.1%)	0.4052[2]
	< 150 g/week	15 (65.2%)	28 (54.9%)	

Categorical variables are represented as frequency (n = absolute number and % percentage). Continuous variables are represented as mean ± standard deviation. 1= based on Kruskal-Wallis test; 2= based on Chi-square test. Values represented as mean±standard deviation

Table S7. Comparisons between patients with obesity or overweight according to total fat percentile and the demographic, lifestyle and biochemical parameters.

VARIABLE		TOTAL FAT PERCENTILE			p-value
		≤36% (N=10)	36%-46% (N=39)	≥46% (n=26)	
Age (Years)		37.60 ± 7.97	38.64 ± 8.31	38.15 ± 9.38	0.9304[1]
Glucose (mg/dL)		89.00 ± 21.86	84.15 ± 11.73	88.64 ± 33.26	0.9022[1]
HbA1c (%)		5.46 ± 0.67	5.47 ± 0.38	5.65 ± 1.28	0.7553[1]
Total cholesterol (mg/dL)		186.70 ± 43.25	183.13 ± 39.96	184.38 ± 32.86	0.9530[1]
HDL (mg/dL)		41.70 ± 10.37	50.28 ± 9.82	47.77 ± 9.73	0.0799[1]
LDL (mg/dL)		89.20 ± 29.07	104.38 ± 32.12	110.35 ± 24.86	0.1442[1]
Triglyceride (mg/dL)		279.10 ± 225.47	124.59 ± 74.25	128.08 ± 60.34	0.0790[1]
Uric acid (mg/dL)		6.38 ± 1.72	5.32 ± 1.52	5.11 ± 1.32	0.0426[1]
Weekly resistance exercise (min)		12.00 ± 37.95	41.79 ± 91.76	28.85 ± 61.47	0.6740[1]
Weekly aerobic exercise (min)		24.00 ± 57.97	90.26 ± 130.45	55.00 ± 100.41	0.2159[1]
Sex	Female	2 (20.0%)	27 (69.2%)	25 (96.2%)	<0.0001[2]
	Male	8 (80.0%)	12 (30.8%)	1 (3.8%)	
Alcohol intake	> 150 g/week	6 (60.0%)	15 (38.5%)	10 (40.0%)	0.4556[2]
	< 150 g/week	4 (40.0%)	24 (61.5%)	15 (60.0%)	

Categorical variables are represented as frequency (n = absolute number and % percentage). Continuous variables are represented as mean ± standard deviation. 1= based on Kruskal-Wallis test; 2= based on Chi-square test. Values represented as mean±standard deviation
